# A simultaneous, high-throughput and sensitive method for analysing 13 neonicotinoids and metabolites in urine using a liquid chromatography–tandem mass spectrometry

**DOI:** 10.1016/j.mex.2023.102129

**Published:** 2023-03-11

**Authors:** Yukiko Nishihama, Shoji F. Nakayama, Tomohiko Isobe

**Affiliations:** Japan Environment and Children's Study Programme Office, National Institute for Environmental Studies, 16-2 Onogawa, Tsukuba, Ibaraki, 305-8506, Japan

**Keywords:** Neonicotinoid, urine, high-throughput analysis, Simultaneous and high-throughput determination of 13 urinary neonicotinoid and metabolite concentrations

## Abstract

A simultaneous, high-throughput and sensitive method for analysing nine neonicotinoid pesticides (NEOs) and four metabolites (NEOms) in urine using a liquid chromatography-tandem mass spectrometry (LC–MSMS) was developed. The method detection limit (MDL) and lowest concentration minimum reporting limit (LCMRL) of the nine NEOs were 0.0013–0.048 ng/ml and 0.0050–0.17 ng/ml, respectively. The MDL and LCMRL of the four NEOms were 0.0052–0.52 ng/ml and 0.011–1.6 ng/ml, respectively. Intermediate precision for the nine NEOs and four NEOms was 7.5–12.5% and 7.4–10.9%, respectively. Accuracy for the nine NEOs and four NEOms was 3.83–5.60% and 3.01–29.2%, respectively. The developed method was applied to analyse urine samples collected from participants of a large-scale birth cohort study, namely, the Japan Environment and Children's Study (JECS).

• The NEO and NEOm concentrations in 100 µl urine samples were analysed using a highly sensitive LC–MSMS.
• Automated solid phase extraction in a 96-well plate was utilised to achieve high-throughput analysis.
• Intermediate precision and accuracy were less than 12.5% and 94.8–99.1%, respectively.

## Graphical abstract

**Figure:**
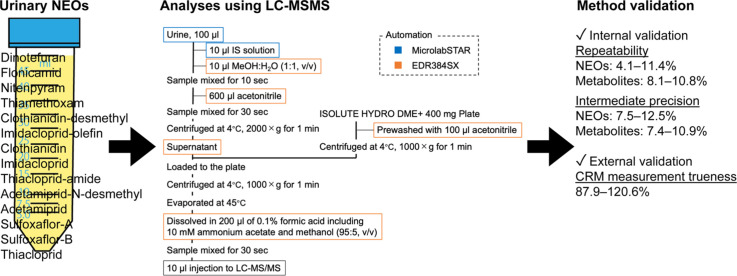

Specifications table

**Table:** 

Subject area:	Environmental Science
More specific subject area:	Environmental health Public health
Name of your method:	Simultaneous and high-throughput determination of 13 urinary neonicotinoid and metabolite concentrations
Name and reference of original method:	N. Suwannarin, T. Prapomontol, T. Isobe, et al., Characteristics of Exposure of Reproductive-Age Farmworkers in Chiang Mai Province, Thailand, to Organophosphate and Neonicotinoid Insecticides: A Pilot Study, Int. J. Environ. Res. Public Health. 17(21) (2020) 7871. https://doi.org/10.3390/ijerph17217871
Resource availability:	(none)

## Method details

### Relevance of this method development

Neonicotinoid pesticides (NEOs) are agonists of neuronal nicotinic acetylcholine receptors [1]. Recently, many epidemiological studies have investigated the relationship between exposure to NEOs and neurodevelopmental delay [2,3]. However, most of these studies used questionnaires or assessed residential proximity to places of NEO use as exposure indices. For more detailed assessment of exposure, biomonitoring, i.e., chemical analysis of biological samples to reveal the internal burden, has been performed. Urinary concentrations can be used to assess NEO exposure. Published methods can capture some NEOs and their metabolites; however, they are not optimised for use in a large-scale cohort study involving hundreds of thousands of subjects. A high-throughput and highly sensitive method is required for the affordable measurements of urinary NEOs in such a study. We modified the currently available method [4] by modifying the SPE method and refining the separation of target compounds in the liquid chromatography, which resulted in the improved method performance including accuracy, precision and method reporting limits.

### Chemicals and reagents

Table 1 shows the neonicotinoids analysed in this study. A standard stock solution mixture of unlabelled NEOs and NEOms, as well as an internal standard (IS) solution including acetamiprid (pyridymethyl-^13^C_6_) (ACE-IS), thiacloprid (pyridymethyl-^13^C_6_) (TCP-IS), sulfoxaflor (2-methyl-^13^C, D_3_; cyanamide-^15^N_2_, ^13^C) (SUL-IS), flonicamid (^18^O; amide-^15^N) (FLN-IS), thiamethoxam (methylene-^13^C; thiazole-^13^C_3_, ^15^N) (THX-IS), dinotefuran (furylmethyl-^13^C_5_) (DIN-IS), clothianidin (methylene-^13^C; thiazole-^13^C_3_, ^15^N) (CLO-IS), imidacloprid (pyridymethyl-^13^C_6_) (IMI-IS), nitenpyram (methyl-^13^C; ethyl-^13^C2;ethenediamine-^15^N_2_) (NIT-IS), acetamiprid-N-desmethyl (acetimidamide-^13^C_3_; amine-^15^N) (dm-ACE-IS), TCP-amid (pyridymethyl-^13^C_6_) (TCP-amid-IS), CLO-desmethyl (guanidine-^13^C; guanidine-1,3-^15^N_2_) (dm-CLO-IS) and IMD-olefin (imidazol-1-^15^N; 2-^13^C; 2-amino-^15^N) (IMI-OF-IS), were purchased from Cambridge Isotope Laboratories, Inc. (Tewksbury, MA, USA). Acetonitrile (purity 99.8%), ammonium acetate (guaranteed reagent), formic acid (purity ≥98.0%), methanol (purity 99.8%) and ultrapure water (total organic carbon ≤15 ppb) were used for this analysis.

**Table 1:** List of analytes.

Analyte	IUPAC name	MW (g/mol)	Acronym	CAS number
Acetamiprid	N-[(6-chloropyridin-3-yl)methyl]-N'-cyano-N-methylethanimidamide	222.67	ACE	160430-64-8
Thiacloprid	[3-[(6-chloropyridin-3-yl)methyl]-1,3-thiazolidin-2-ylidene]cyanamide	252.72	TCP	111988-49-9
Sulfoxaflor	[methyl-oxo-[1-[6-(trifluoromethyl)pyridin-3-yl]ethyl]-lambda6-sulfanylidene]cyanamide	277.27	SUL	946578-00-3
Flonicamid	*N*-(cyanomethyl)-4-(trifluoromethyl)pyridine-3-carboxamide	229.16	FLN	158062-67-0
Thiamethoxam	(*NE*)-*N*-[3-[(2-chloro-1,3-thiazol-5-yl)methyl]-5-methyl-1,3,5-oxadiazinan-4-ylidene]nitramide	291.72	THX	153719-23-4
Dinotefuran	1-methyl-2-nitro-3-(oxolan-3-ylmethyl)guanidine	202.21	DIN	165252-70-0
Clothianidin	1-[(2-chloro-1,3-thiazol-5-yl)methyl]-3-methyl-2-nitroguanidine	249.68	CLO	210880-92-5
Imidacloprid	(*NE*)-*N*-[1-[(6-chloropyridin-3-yl)methyl]imidazolidin-2-ylidene]nitramide	255.66	IMI	138261-41-3
Nitenpyram	(*E*)-1-*N*'-[(6-chloropyridin-3-yl)methyl]-1-*N*'-ethyl-1-*N*-methyl-2-nitroethene-1,1-diamine	270.71	NIT	150824-47-8
Acetamiprid-N-desmethyl	*N*'-[(6-chloropyridin-3-yl)methyl]-*N*-cyanoethanimidamide	208.65	dm-ACE	190604-92-3
Thiacloprid-amide	(*Z*)-[3-[(6-chloropyridin-3-yl)methyl]-1,3-thiazolidin-2-ylidene]urea	270.74	TCP-amid	676228-91-4
Clothianidin-desmethyl	2-[(2-chloro-1,3-thiazol-5-yl)methyl]-1-nitroguanidine	235.65	dm-CLO	135018-15-4
Imidacloprid-olefin	*N*-[1-[(6-chloropyridin-3-yl)methyl]imidazol-2-yl]nitramide	253.64	IMI-OF	115086-54-9

### Sample preparation

**Fig. 1:**
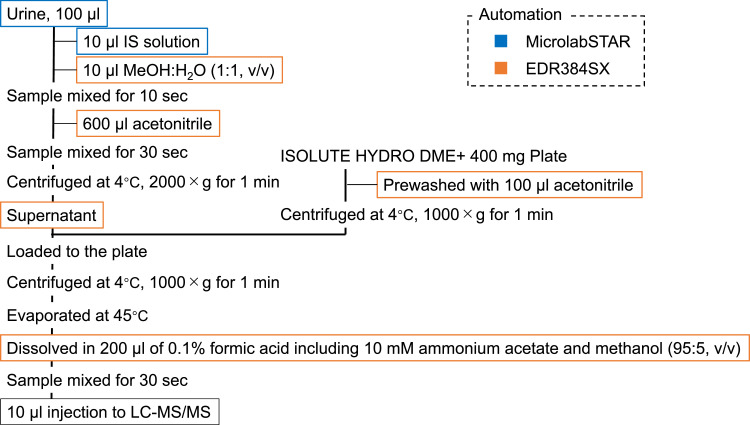
Flowchart of sample preparation.

The sample preparation method was based on our previous study [4] and modified to achieve high-throughput analysis. The major modification was automation of sample preparation using Microlab STAR (Hamilton Company, Reno, NV, USA) and EDR-384SX (BIOTEC Co., Ltd., Tokyo, Japan). First, 10 µl of IS solution and 10 µl of 50% methanol in water was added to 100 µl of each urine sample. Then, 600 µl of acetonitrile was added to precipitate proteins and centrifuged at 4°C, 2000 × g for 1 minute. An ISOLUTE® HYDRO DME+ 400 mg plate (Biotage, Uppsala, Sweden) was prewashed with 100 µl of acetonitrile and centrifuged at 4°C, 1000 × g for 1 minute. The supernatants of samples were loaded onto the plate and centrifuged at 4°C, 1000 × g for 1 minute. The samples were evaporated to dryness with a centrifugal vacuum concentrator in combination with the TurboVap 96-well system (Biotage) at 45°C. Residues were dissolved with 200 µl of 5% methanol in 0.1% formic acid and 10 mM ammonium acetate and mixed for 30 seconds. From the final sample, 10 µl of each eluate was injected into a liquid chromatograph–tandem mass spectrometer (LC–MSMS) (Fig. 1).

### Instrument analysis and calculations

**Fig. 2:**
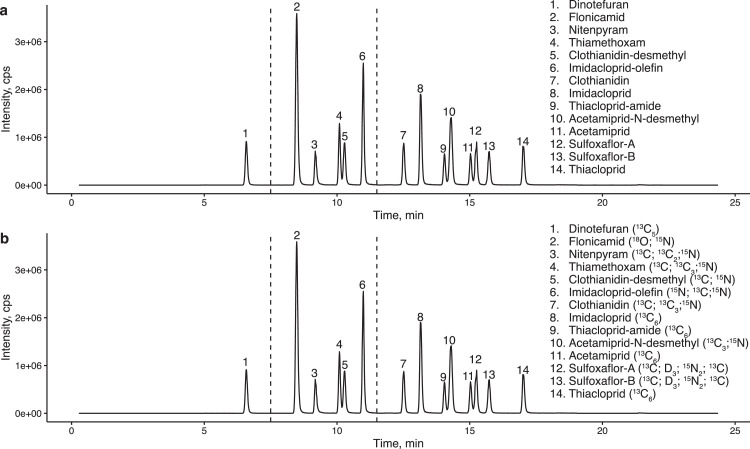
Typical chromatograms of target compounds. a: native standard solution, b: stable isotope labeled internal standard solution.

The Nexera X2 system (Shimadzu Corporation, Kyoto, Japan) was used for separation. A Triple Quad^TM^ 6500 mass spectrometer (AB Sciex LLC, MA, USA) was used to detect and quantify target analytes. The MSMS system was operated using electrospray ionisation positive mode, and multiple reaction monitoring was performed. An ACQUITY UPLC HSS T3 (100 Å, 2.1 mm × 100 mm, 1.8 µm; Waters, Ireland) was used as an analytical column. The LC and MSMS parameters are listed in Table 2, Table 3, Table 4. LC–MSMS run time was 24.5 minutes per sample. Multiple LC–MSMS systems were used parallelly to accelerate the analysis. The total analysis time was estimated to be 26.5 minutes per sample. Typical chromatograms are shown in Fig. 2.

**Table 2:** Liquid chromatography conditions

	Time (minutes)	Mobile phase A (%): 0.1% formic acid in 10 mM ammonium acetate	Mobile phase B (%): Methanol
Programme of mobile phase	0.01	95	5
	4.00	95	5
	18.00	65	35
	18.50	2	98
	20.50	2	98
	20.51	95	5
	24.50	95	5
Setting of valves switching		Position	
	0.00–5.00	Waste	
	5.00–19.00	Load to tandem mass spectrometer	
	19.00–24.50	Waste	
Flow rate	0.4 ml/min		
Injection volume	10 µl		
Solvent for needle wash	Methanol:water = 7:3		
Temperature of column oven	50°C		
Temperature of autosampler	4°C		
Run time	24.5 minutes

**Table 3:** Mass parameters and LC retention time

	Target ion (m/z)		Qualifier ion (m/z)		Declustering potential (V)		Collision energy (V)		Dwell time (milliseconds)	Retention time (min)
	Precursor	Product	Precursor	Product	Target	Qualifier	Target	Qualifier		
ACE	223.2	126.0	223.2	99.0	50	50	28	53	30	15.0
TCP	253.0	126.0	253.0	90.1	40	40	27	50	30	17.0
SUL-A	174.0	153.9	174.0	104.1	40	40	25	37	30	15.3
SUL-B	174.0	153.9	174.0	104.1	40	40	25	37	30	15.7
FLN	230.1	203.0	230.1	174.1	80	80	22	23	40	8.5
THX	292.0	211.0	292.0	181.1	30	30	17	30	40	10.1
DIN	203.0	114.2	203.0	157.0	35	35	18	11	200	6.6
CLO	249.9	169.2	249.9	132.0	35	35	17	21	30	12.5
IMI	256.3	209.1	256.3	175.2	35	35	21	26	30	13.2
NIT	271.2	237.1	271.2	99.1	60	60	25	68	40	9.2
dm-ACE	208.9	90.1	208.9	99.1	40	40	42	50	30	14.3
TCP-amid	271.1	126.0	271.1	228.0	60	60	32	19	30	14.1
dm-CLO	236.1	132.0	236.1	155.1	30	30	17	17	40	10.3
IMI-OF	254.1	204.9	254.1	171.1	50	50	20	24	40	11.0
ACE-IS	229.0	132.2	-	-	50	-	30	-	30	15.0
TCP-IS	259.0	132.1	-	-	40	-	35	-	30	17.0
SUL-A-IS	178.2	157.1	-	-	40	-	26	-	30	15.2
SUL-B-IS	178.2	157.1	-	-	40	-	26	-	30	15.7
FLN-IS	233.1	206.2	-	-	80	-	25	-	40	8.5
THX-IS	297.0	216.1	-	-	30	-	20	-	40	10.1
DIN-IS	208.1	132.3	-	-	35	-	18	-	200	6.6
CLO-IS	255.0	174.0	-	-	35	-	20	-	30	12.5
IMI-IS	262.0	215.1	-	-	35	-	26	-	30	13.2
NIT-IS	276.0	242.3	-	-	60	-	26	-	40	9.2
dm-ACE-IS	214.1	90.1	-	-	40	-	45	-	30	14.3
TCP-amid-IS	277.0	234.1	-	-	60	-	21	-	30	14.0
dm-CLO-IS	241.1	133.9	-	-	30	-	21	-	40	10.3
IMI-OF-IS	259.1	210.2	-	-	50	-	23	-	40	11.0

ACE, acetamiprid; TCP, thiacloprid; SUL, sulfoxaflor; FLN, flonicamid; THX, thiamethoxam; DIN, dinotefuran; CLO, clothianidin; IMI, imidacloprid; NIT, nitenpyram; IS, stable isotope labelled internal standard; dm-ACE, acetamiprid-N-desmethyl; TCP-amid, thiacloprid-amide; dm-CLO, clothianidin-desmethyl; IMI-OF, imidacloprid-olefin; LC, liquid chromatography.

**Table 4:** Ion source and collision cell conditions

Parameter	Setting
IonSpray voltage (V)	5500
Heating gas temperature (°C)	450
Nebulizer gas (psi)	50
Heating gas (psi)	60
Curtain gas flow (psi)	20
Collision gas pressure (psi)	11

The calibration range is shown in Table 5*.* Sulfoxaflor (SUL) was a diastereomer. The two isomers were quantified separately as SUL-A and SUL-B. All samples that fell outside the calibration range were reanalysed following further dilution.

**Table 5:** Ranges of calibration curve

	Concentrations (ng/ml)									IS	IS concentrations (ng/ml)
	C0	C1	C2	C3	C4	C5	C6	C7	C8		
ACE	0	0.0050	0.010	0.020	0.050	0.10	0.20	0.50	1.0	ACE-IS	0.20
TCP	0	0.0050	0.010	0.020	0.050	0.10	0.20	0.50	1.0	TCP-IS	0.20
SUL-A SUL-B	0	0.0050	0.010	0.020	0.050	0.10	0.20	0.50	1.0	SUL-A-IS	0.40
	0	0.0050	0.010	0.020	0.050	0.10	0.20	0.50	1.0	SUL-B-IS	0.40
FLN	0	0.10	0.20	0.40	1.0	2.0	4.0	10	20	FLN-IS	8.0
THX	0	0.020	0.040	0.080	0.20	0.40	0.80	2.0	4.0	THX-IS	0.80
DIN	0	0.050	0.10	0.20	0.50	1.0	2.0	5.0	10	DIN-IS	2.0
CLO	0	0.050	0.10	0.20	0.50	1.0	2.0	5.0	10	CLO-IS	2.0
IMI	0	0.050	0.10	0.20	0.50	1.0	2.0	5.0	10	IMI-IS	2.0
NIT	0	0.050	0.10	0.20	0.50	1.0	2.0	5.0	10	NIT-IS	2.0
dm-ACE	0	0.050	0.10	0.20	0.50	1.0	2.0	5.0	10	dm-ACE-IS	4.0
TCP-amid	0	0.010	0.020	0.040	0.10	0.20	0.40	1.0	2.0	TCP-amid-IS	0.40
dm-CLO	0	0.20	0.40	0.80	2.0	4.0	8.0	20	40	dm-CLO-IS	16
IMI-OF	0	1.0	2.0	4.0	10	20	40	100	200	IMI-OF-IS	40

ACE, acetamiprid; TCP, thiacloprid; SUL, sulfoxaflor; FLN, flonicamid; THX, thiamethoxam; DIN, dinotefuran; CLO, clothianidin; IMI, imidacloprid; NIT, nitenpyram; dm-ACE, acetamiprid-N-desmethyl; TCP-amid, thiacloprid-amide; dm-CLO, clothianidin-desmethyl; IMI-OF, imidacloprid-olefin; IS, stable isotope labelled internal standard.

### Method validation/quality control (QC)

A pooled urine sample was prepared using maternal samples collected from volunteers to create QC samples. Levels of analytes varied in actual samples, so we tried to create the QC sample to reflect such concentrations. The QC samples had the concentration of dinotefuran around 2 ng/ml. To double the concentration, NEOs and NEOms were fortified such that the concentrations of ACE, TCP, SUL-A, SUL-B, FLN, THX, DIN, CLO, IMI, NIT, dm-ACE, TCP-amid, dm-CLO and IMI-OF were 0.5, 0.5, 0.5, 0.5, 10, 2.0, 5.0, 5.0, 5.0, 5.0, 5.0, 1.0, 20 and 100 ng/ml, respectively. The QC samples were subjected to the same procedure as the unknown samples, with five replicates analysed in each analytical sequence. The lowest concentration minimum reporting limit (LCMRL) was calculated following the U.S. Environmental Protection Agency's instructions [5] and summarised in Table 6. The method detection limits (MDLs) in the current study were comparable with those in a previous study [6], except that the MDLs of ACE, DIN and CLO were 2–100 times lower than those obtained using previous methods [6,7].

**Table 6:** Method performance characteristics

	Linearity R^2^	MDL (ng/ml)	LCMRL (ng/ml)	Accuracy (%)	IS recovery (%)	Repeatability (%)	Intermediate precision (%)
ACE	0.991	0.0027	0.0077	95.7	98.7	4.1	12.5
TCP	0.992	0.0040	0.0079	97.5	97.8	10.6	9.8
SUL-A	0.991	0.0027	0.0069	99.1	92.7	8.5	8.2
SUL-B	0.990	0.0030	0.0061	94.8	96.4	9.5	8.6
FLN	0.994	0.048	0.17	96.4	91.9	10.0	9.1
THX	0.992	0.017	0.034	98.0	96.6	9.8	8.8
DIN	0.995	0.022	0.070	96.1	90.1	8.7	7.7
CLO	0.991	0.037	0.077	95.4	102	11.4	10.0
IMI	0.991	0.031	0.076	97.3	103	9.9	8.7
NIT	0.991	0.043	0.071	97.5	101	7.4	7.5
dm-ACE	0.990	0.040	0.072	95.9	97.1	8.3	7.4
TCP-amid	0.990	0.0078	0.018	97.3	99.7	10.8	9.6
dm-CLO	0.990	0.17	0.33	97.3	89.7	8.1	7.4
IMI-OF	0.990	0.52	1.6	97.5	97.5	10.8	10.9

ACE, acetamiprid; TCP, thiacloprid; SUL, sulfoxaflor; FLN, flonicamid; THX, thiamethoxam; DIN, dinotefuran; CLO, clothianidin; IMI, imidacloprid; NIT, nitenpyram; dm-ACE, acetamiprid-N-desmethyl; TCP-amid, thiacloprid-amide; dm-CLO, clothianidin-desmethyl; IMI-OF, imidacloprid-olefin; MDL, method detection limit; LCMRL, lowest concentration minimum reporting limit; IS, stable isotope labelled internal standard; R^2^, coeficient of determination.

The eight-point calibration curves showed coefficients of determination (R^2^) higher than 0.990. Repeatability and intermediate precision were determined based on ISO 5725:1994 and 27148:2010, with QC sample measurements (n = 100). QC for day-to-day analysis was determined using a Shewhart control chart ($\bar{X}$-R control chart) according to ISO 7870. Intermediate precision was less than 12.5% (Table 6). Overall accuracy was 94.8–99.1%. The percent recoveries of target compounds were calculated by fortifying a QC sample and measuring these concentrations in 20 replicates. Overall IS percent recoveries were 90.1–103.4%.

For external validation, five certified reference material (CRM) samples (NMIJ CRM 7408-a) with certified concentrations (expanded uncertainty concentrations, SD) at 25°C (1.0125 g/cm^3^ specific gravity) of 1.40 (0.27), 1.36 (0.31), 0.19 (0.06) and 1.34 (0.47) ng/ml for ACE, CLO, TCP and THX, respectively, were analysed in nine replicates. All measured concentrations fell within the certified ranges (Table 7).

**Table 7:** Results of the measurements of the certified reference material (CRM), NMIJ CRM 7408-a

		Mean (SD) [ng/ml]			
	n	ACE	CLO	TCP	THX
Instrument 1	5	1.48 (0.054)	1.23 (0.025)	0.20 (0.0050)	1.49 (0.034)
Instrument 2	5	1.54 (0.044)	1.35 (0.036)	0.20 (0.0064)	1.59 (0.044)
Instrument 3	10	1.50 (0.046)	1.26 (0.050)	0.20 (0.0063)	1.50 (0.061)
Instrument 4	5	1.52 (0.033)	1.27 (0.023)	0.20 (0.0086)	1.55 (0.024)
Instrument 5	20	1.44 (0.029)	1.24 (0.048)	0.20 (0.0064)	1.49 (0.049)
Reference value^1^	-	1.40 (0.27)	1.36 (0.31)	0.19 (0.06)	1.34 (0.47)

ACE, acetamiprid; TCP, thiacloprid; THX, thiamethoxam; CLO, clothianidin; SD, standard deviation; NMIJ, National Metrology Institute of Japan.

1 Certified concentration (expanded uncertainty concentrations) [ng/ml]: a unit of mass fraction of certified values was converted into concentration values using the value of specific gravity (1.0125 g/cm^3^).

## Ethics statements

Not applicable.

## CRediT authorship contribution statement

**Yukiko Nishihama:** Conceptualization, Formal analysis, Writing – original draft, Visualization. **Shoji F. Nakayama:** Methodology, Software, Writing – review & editing, Supervision, Project administration. **Tomohiko Isobe:** Methodology, Investigation, Writing – review & editing.

## Declaration of competing interest

The authors declare that they have no known competing financial interests or personal relationships that could have appeared to influence the work reported in this paper.

## Data Availability

Data will be made available on request.
